# Percutaneous Treatment of Geriatric Distal Tibia and Fibula Fractures With a Poor Soft Tissue Envelope – A Case Series

**DOI:** 10.1177/21514593261446750

**Published:** 2026-06-16

**Authors:** Pedram Rajabifard, Joshua Booth, Samuel Hookway, Richard Kjar, Nathan Donovan, Anton Lambers

**Affiliations:** 1 72537 Orthopaedic Department, Northeast Health Wangaratta, Wangaratta, Victoria, Australia; 2 3487 Orthopaedic Department, Barwon Health, Geelong, Victoria, Australia; 3 Medical School, Division of Surgery, The University of Western Australia, Perth, Western Australia, Australia

**Keywords:** ankle, fracture, geriatric, percutaneous, fixation

## Abstract

**Purpose:**

Geriatric distal tibia and fibula fractures are typically treated with open reduction and internal fixation (ORIF) or prolonged cast immobilisation. These methods predispose to wound complications and delayed weight-bearing. Percutaneous fixation can offer a stable construct allowing for early weight-bearing while minimising wound complications. This case series details the management and outcomes of patients over 65 with distal tibia and fibula fractures and poor soft tissue envelope treated with percutaneous fixation.

**Methods:**

A prospective observational study was conducted at two Victorian orthopaedic units, identifying patients over 65 years of age who underwent percutaneous distal tibia or fibula fracture fixation. Patient selection considered soft tissue quality and overall health. Antegrade tibial intramedullary nails and retrograde percutaneous screws were used. Surgery proceeded without delay for swelling or anticoagulation cessation. Clinical notes and radiographs were analysed for premorbid health, mobility status, injury mechanism and classification, fixation methods, post-operative mobility, and treatment complications.

**Results:**

The study identified 13 ankles in 11 patients (average age 82) across two sites. Fractures included 4 trimalleolar, 5 bimalleolar, 1 lateral malleolar, and 3 distal tibia and fibula diaphyseal. Three fractures were open. Median time to operation was 3 days. Six patients were allowed immediate weight-bearing as tolerated. Median post-operative stay was 5 days. One deep infection required removal of a suture-button syndesmosis device and two minor wound breakdowns (distant to surgical site) were managed non-operatively with dressings.

**Conclusion:**

Percutaneous distal tibia and fibula fracture fixation appears to be a viable alternative to ORIF in geriatric patients with poor soft tissue envelope, potentially offering reduced complications and earlier mobilisation.

**Level of evidence:**

Level IV.

## Introduction

The incidence and severity of geriatric distal tibia and fibula injuries is increasing.^1,2^ These injuries primarily occur because of low-energy trauma such as a fall from standing height. There remains uncertainty over the optimal management of geriatric distal tibia and fibula fractures particularly in the context of a poor soft tissue envelope.^3-8^

The crux of the issue lies in the balance between minimizing soft tissue dissection yet maximizing stability to allow for early weightbearing. Creating a stable ankle joint that allows early rehabilitation is more important than anatomic reduction in this age group where post traumatic osteoarthritis is less of a concern than deconditioning.^9^

Across all age groups, open reduction internal fixation (ORIF) remains the standard of care for unstable ankle fractures.^2,10^ In the elderly this carries an all-cause complication rate of 35 to 40%.^4,6^ Open surgery may need a period of elevation for swelling to subside, further delaying mobilisation.^2,6-8,11,12^ Alternative treatments to mitigate these risks include casting, hindfoot nailing and minimally invasive fixation.^5,11,13^

Minimally invasive surgical techniques in this context have reduced complication rates.^2,5^ Percutaneous medial malleolar fixation is not a novel concept, however distal fibula fractures usually undergo ORIF. Fixation of the distal fibula with intramedullary (IM) nails or screws allows for smaller incisions with reduced soft tissue dissection.^5^ Decreased time to surgery and earlier discharge in patients undergoing minimally invasive ankle fixation have also been demonstrated.^2^ Many operating theatres do not stock fibular nails routinely and these are more costly, however cannulated screws are usually readily available.

Systematic reviews of fibula IM screw fixation report high rates of union and high-quality reduction.^2,5,13^ Patient outcome measures are also encouraging, with up to 88% of AOFAS ankle hindfoot scores being good or excellent at 1 year follow up.^10^ The all-cause complication rates with IM screw fixation is low at 5 to 9%.^2,7^

There is a paucity of data researching minimally invasive management of the geriatric patient with an unstable distal tibia and fibula fractures. In our opinion percutaneous fixation is ideal in these high-risk patients. When compared to ORIF it carries the benefit of early surgery and stable fixation without the additional risks of a large exposure. Compared to hindfoot nailing there is no violation of the tibio-talar joint surfaces, no plantar wound, ankle range of motion is maintained and immediate weightbearing is a viable option. In contrast to cast immobilization the patient is allowed to bear weight 6 to 8 weeks earlier than usual, without additional risk to the wound.^14^

The primary aim of this study was to determine the safety profile of a technique for percutaneous fixation of distal tibia and fibula fractures primarily using large caliber (6.5 mm) cannulated screws. We hypothesized that small incisions with robust fixation would facilitate a low wound complication rate, early weightbearing and a reduced length of stay.

## Methods

A prospective observational study of geriatric patients undergoing percutaneous fixation of traumatic distal tibia and fibula fractures was undertaken at two centers in Victoria, Australia (Northeast Health Wangaratta and Barwon Health) over a 12-month period. Ethical approval for this study was obtained from Northeast Health Wangaratta Research Office (QA/102245/NEHW-2023-396421). This study was reported in accordance with the STROBE guidelines.

**Figure 1.**
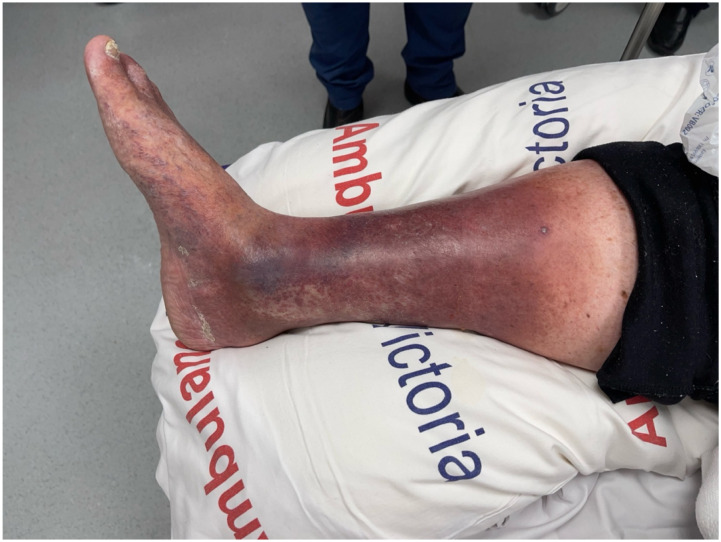
Patient skin (Case 9) showing severe venous eczema in a patient with poorly controlled diabetes

Patients aged over 65 years old with a distal tibia and or fibula fracture were prospectively identified. Patient selection was at surgeon discretion, with the fundamental underlying factor being poor tissue quality of the skin as a direct subjective observation. Whilst Tscherne classification was not recorded routinely at these institutions, considerations that encouraged the lead surgeons to perform this surgery were the presence of paper-thin skin, open injuries, peripheral neuropathy, peripheral vascular disease, severe venous eczema, diabetes, older age and general frailty. A patient example deemed appropriate is shown in Figure 1. There were no pathological fractures or pre-existing active local infection in these patients. Patients with isolated syndesmotic injuries (e.g. Maisonneuve type injuries) were not included. There were no other strict exclusion criteria. Clinical Frailty Scores were calculated on admission and on discharge.

All patients received intravenous cefazolin prior to skin incision, 2 grams if less than 120 kilograms and 3 grams if more than 120 kilograms as per local hospital policy. Open fractures received intravenous cefazolin 8 hourly upon admission until surgery and for a total of 24 hours post-surgery. No oral courses of antibiotics were prescribed to any patient. There were no contaminated open wounds in our series. Patients were strived to be operated on as soon as medically ready, without surgical delays due to soft tissue considerations.

Surgical technique and implant selection was fracture- and surgeon-dependent but generally aimed to use as little dissection as able to allow fracture and/or joint stability. Reduction of fragments was performed by closed means or by using reduction clamps or instruments through percutaneous incisions. Large caliber (6.5 mm) cannulated screw fixation was the mainstay of implant selection. We felt the larger diameter and thread size improved bony purchase and allowed us to use it similar to an intramedullary nail for strength and load bearing. We accepted threads being too wide in the medial-lateral direction. All screws were first drilled then tapped through the entire screw length to reduce risk of complications such as fracture in osteoporotic bone. Stress radiographs were obtained intraoperatively to assess syndesmosis integrity post fixation and additional fixation was employed as necessary. No closed fractures were opened for reduction. Post-operative restrictions and immobilization were decided by the surgeon for each case.

Follow up was unchanged from routine practice and typically involved at least a wound assessment at 2 weeks and a clinical review and radiograph at 6 weeks.

Data collected included patient demographics, fracture characteristics and other treatment information obtained from the medical records. Where a patient used different gait aids for different scenarios, their least supportive gait aid was recorded. Operation duration was captured from theatre data as time from first incision to completion of procedure. For bilateral cases this figure was halved. Patients were assessed for complications at all points of follow up. Complications assessed included wound dehiscence or infection, loss of position or fixation and medical complications. Clinical photographs were taken with consent from select patients to supplement the data presented.

Descriptive statistics were performed using Microsoft Excel (Version 16.106.2, Microsoft Corporation, Redmond, Washington, USA). Parametric data was described using mean and standard deviation, non-parametric data was described using median and interquartile range.

## Results

A total of 13 fractures (11 patients) that met the inclusion criteria were identified between May 2020 to October 2023. The senior author (AL) performed 10 of the 13 procedures.

The patient demographics are summarized in Table 1 with fracture characteristics in Table 2. The injury pattern and corresponding implant selection is shown in Table 3. All injuries were sustained in low energy trauma. Treatment and post-operative instructions are shown in Table 4. One patient required additional suture tape fixation for management of their syndesmosis injury. The patients that were allowed to weightbear as tolerated (WBAT) post operatively all commenced mobilizing with the help of an optional gait aid for support which was weaned as soon as it was safe to do so from a balance and falls perspective. Patient level data is available in Supplement A.

**Table 1.** Patient Demographics

Demographics	​
Age (mean ± Standard Deviation, range)	82±9 (67-92) years
Sex	​
Male	2
Female	9
Comorbidities (median, Interquartile range, range)	8 (IQR 6, range 2-11)
Premorbid residential status	​
Home	8
Aged care facility	3
Premorbid mobility	​
Nil aids	3
Single point stick	1
4 wheel walker	7
Clinical Frailty Score on admission (median, Interquartile range, range)	4 (IQR 4–5; range 3–6)

**Table 2.** Fracture Characteristics

Fracture pattern	
Trimalleolar Fracture	4
Medial + Lateral Malleoli	3
Distal Tibia and Fibula	3
Isolated Lateral Malleolus	1
Posterior + Medial Malleoli	1
Posterior + Lateral Malleoli	1
Ankle Joint on Presentation	
Dislocated	3
Subluxated	3
Enlocated	4
N/A (distal tibia/fibula)	3
Skin Integrity	
Open	3
Closed	10

**Table 3.** Case Specific Fracture Patterns and Fixation Methods

Case	Fracture pattern	Implant used
1	Distal Tibia/fibula	6.5mm FT CS for MM
		3.5mm FT CS for LM
2	Trimalleolar	6.5mm FT CS for MM
		6.5mm FT CS for LM
3(R)	PM + MM	4.0mm PT CSx2 for MM
		6.5mm FT CS for LM
3(L)	PM + LM	6.5mm FT CS for LM
4	MM + LM	6.5mm FT CS for LM
		6.5mm PT CS for MM
5(R)	Distal tibia/fibula	Tibial IMN
		6.5 FT CS for LM
5(L)	Distal tibia/fibula	Tibial IMN
		6.5 FT CS for LM
6	MM + LM	6.5mm FT CS for MM
		6.5mm FT CS for LM
7	Trimalleolar	6.5mm FT CS for MM
		6.5mm FT CS for LM
		4mm PT CS for PM
8	Trimalleolar	6.5mm FT CS for MM
		6.5mm FT CS for LM
9	LM	6.5mm FT CS for LM
		Suture button for syndesmosis
10	MM + LM	6.5mm FT CS for LM
		4mm PT CS for MM
11	MM + LM	6.5mm FT CS for LM
		6.5mm PT CS for MM

Key: CS (cannulated screw), IMN (intramedullary nail), FT (fully threaded), PT (partially threaded), MM (medial malleolus), LM (lateral malleolus), PM (posterior malleolus).

**Table 4.** Treatment and Outcomes

Median time to operation (IQR, range)	3 (2, 0-13) days
Median operation duration (IQR, range)	50 (34, 25-210) min
Post operative immobilisation	
None	4
Functional walking (CAM) boot	5
Cast	4
Post operative weightbearing instructions	​
WBAT	8
Touch WB	1
NWB	4
Patient mobility on final review	​
Unaided	4
Frame	4
Sara Steady	3
Length of stay (median, IQR, range)	5 (5, 0-20) days
Patient discharge destination	​
Home	2
Rehabilitation facility	6
Nursing home	3
Clinical Frailty Score on discharge (median, Interquartile range, range)	5 (IQR 4–6; range 3–7)

One patient sustained a small medial malleolar pressure area (distant from surgical incisions) from rubbing on a functional walking boot that was managed with wound care and dressings, noted at the 2 week follow up appointment. The same patient had a deep infection of their suture tape necessitating reoperation for removal of suture tape and debridement 11 weeks post operation. The fracture had united at this point. Post operatively, the surgical wound healed without further complication. One patient had a small area of slough at the site of their open skin wound that was managed with wound care and dressings. There were no surgical site infections, surgical wound breakdowns or loss of fixation. One post operative death occurred 36 days post-operation from multi-organ failure unrelated to surgery in a separate admission.

Length of follow up ranged between 3 days and 268 days, with an average of 60 days.

**Figure 2.**
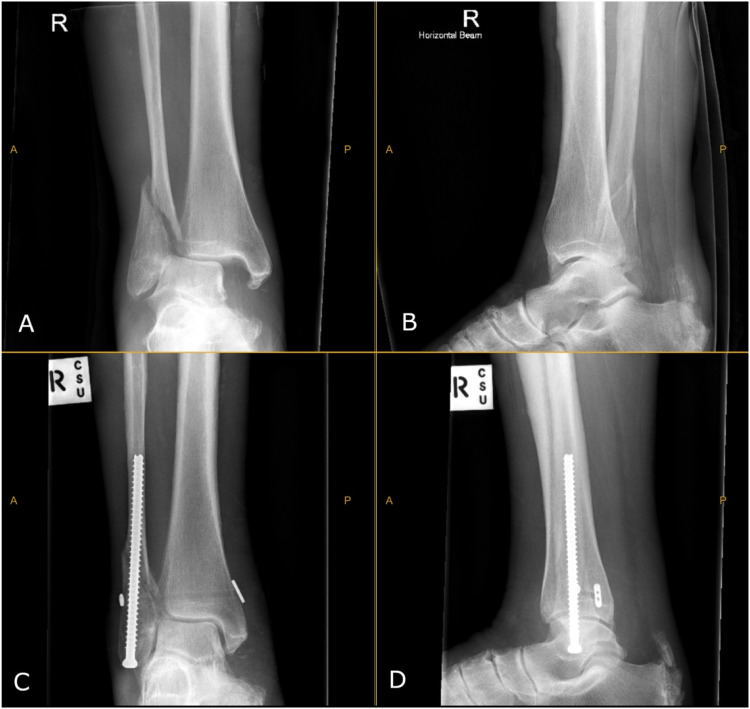
Radiographs of Case 9. Preoperative radiographs (A = mortise view; B = lateral view) demonstrate a subluxated ankle joint with a spiral distal fibula fracture. Postoperative radiographs at 6 weeks post operation (C = mortise view; D = lateral view) demonstrate a uniting fracture fixed with a 6.5mm cannulated screw in the fibula and a syndesmotic suture button

**Figure 3.**
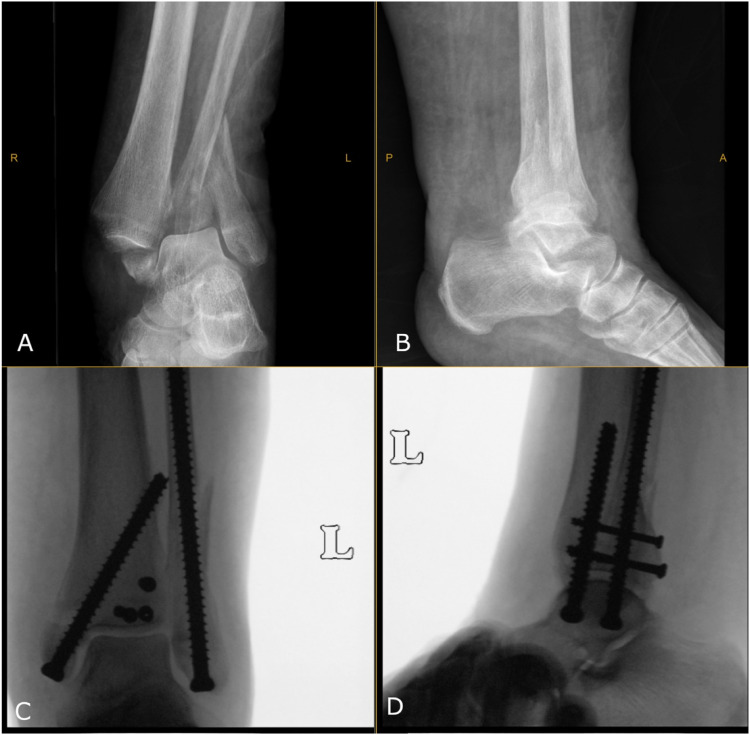
Radiographs of Case 7. Preoperative radiographs (A = anteroposterior view; B = lateral view) demonstrate a dislocated ankle joint with a trimalleolar fracture. Intraoperative image intensifier imaging (C = mortise view; D = lateral view) demonstrating excellent reduction and fixation with combination of 6.5mm and 4mm cannulated screws

Case 9 is shown in Figures 1 and 2. A second case (Case 7) is shown in Figure 3.

## Discussion

We present an observational study of percutaneous fixation of geriatric distal tibia and fibula fractures in patients with a poor soft tissue envelope.

Demographically, the patients in our study were older and had a poorer medical baseline compared to study cohorts in current literature.^6,12^ Our rate of open injuries of 23% was is in keeping with the other rates reported in the geriatric population.^1^

There were 3 complications in 2 patients. One complication was deep infection requiring removal of implant (suture tape) and debridement. Two complications were skin-related and remote to surgical incisions. This is a modest improvement on the 35 to 40% all-cause (predominantly wound) complication rate in geriatric patients treated with ankle ORIF.^4,6^ The complication rates were higher than those reported in other percutaneous screw studies by Jain et al and Loukachov et al, which is likely explained by the sub-selection of comorbid surgical candidates in our series.^2,7^

Larger caliber screws do not always confer improved stability, as demonstrated by syndesmosis screw fixation studies showing no biomechanical advantage of 4.5mm screws over 3.5mm screws.^15^ However, the use of 6.5mm screws allowed for improved cortical engagement especially within the fibula, with no features of cut out on any follow-up radiographs noted.

The non-rigid construct of the large caliber screws inherently increases the risk of loss of position or hardware failure. However, we believe the patient and economic benefits of early return to function as a result of early weight-bearing in a cohort of patients who experience rapid decline when made immobile exceed the risks of a loss of position, which was not noted in our patient cohort. This was demonstrated with most patients being allowed to WBAT and this instruction became the standard as the authors grew experience with the technique. Of the two soft tissue complications, one had been allowed to weight bear as tolerated immediately post operatively and one was made non-weightbearing for 6 weeks. These findings are consistent with those of a 2024 Cochrane review of rehabilitation post ankle fractures in adults that found no statistically significant difference in wound dehiscence, wound healing complications or low grade infections in patients who were allowed early weightbearing.^16^

Our case series had several limitations. A low number of patients were eligible for percutaneous fixation due to selection only of elderly patients with severe soft tissue problems and significant comorbidities. The standard treatment for well patients with good skin remains ORIF. Study size included all patients this rare technique was performed on over the study period. Sample size calculation was not performed as a result as this was a prospective study of all patients, with patient numbers being defined temporally and not by power. The patients were heterogenous with regards to their fracture pattern and fixation method which demonstrates the variety of injuries that occur. This combined with a small cohort and lack of functional outcome assessment makes it difficult to draw definitive conclusions about the results and their broader application. Overall, the follow up of our cases was short. In some instances, longer follow up was not possible after the patient was transferred back to their aged care facility. The shorter follow up will underestimate the final mobility outcome of our patients. However, given the potential for significant improvement in both health economics and patient rehabilitation the early results of this technique were felt important to disseminate.

It is possible to miss complications that present to other institutions however a strength of the study was that the two recruiting sites were both regional centers in towns where there is only one public hospital for patients to return to. Union was not assessed as final follow up was typically 6 weeks or less.

Distal tibia and fibula fractures in geriatric patients with poor soft tissue envelopes are a common challenge for many orthopaedic departments. Future studies with larger patient cohorts, a more homogenous injury pattern and longer follow up would be of benefit to confirm the preliminary results we have seen in our study.

## Conclusion

In summary we have demonstrated that percutaneous fixation of distal tibia and fibula fractures in elderly patients with a poor soft tissue envelope offers an effective alternative with a favourable complication profile and an ability to permit both early surgery and weightbearing. This study provides the impetus for larger comparative studies to build its evidence base.

## Supplemental Material

Supplemental Material - Percutaneous Treatment of Geriatric Distal Tibia and Fibula Fractures With a Poor Soft Tissue Envelope – A Case Series Supplemental Material for Percutaneous Treatment of Geriatric Distal Tibia and Fibula Fractures With a Poor Soft Tissue Envelope – A Case Series by Pedram Rajabifard, Joshua Booth, Samuel Hookway, Richard Kjar, Nathan Donovan & Anton Lambers, in Geriatric Orthopaedic Surgery & Rehabilitation.

## Data Availability

All patient level data utilised in this study are available in Supplement A.*
