# Supplementary material for: Percutaneous Treatment of Geriatric Distal Tibia and Fibula Fractures With a Poor Soft Tissue Envelope – A Case Series
Source: Geriatr Orthop Surg Rehabil. 2026 Jun 16;17:21514593261446750. doi: 10.1177/21514593261446750 (PMC13287419; doi:10.1177/21514593261446750)
Supplement: Supplemental Material - Percutaneous Treatment of Geriatric Distal Tibia and Fibula Fractures With a Poor Soft Tissue Envelope – A Case Series [file sj-pdf-1-gos-10.1177_21514593261446750.pdf]

Supplement A

Case	Age	S	Days to OT	S	Pattern	Ankle stability	Open v Closed	Hardware	Operation duration	Post op immobilisation	Post op restrictions
1	69	M	0	R	Distal Tibia/fibula	N/A	Open	6.5mm FT CS for MM + 3.5mm FT CS for LM;	210	Cast	NWB
2	93	F	3	L	Trimalleolar	Dislocated	Open	6.5mm FT CS for MM + LM	38	CAM	WBAT
3(R)	76	M	3	R	PM + MM	Enlocated	Closed	4.0mm PT CSx2 for MM; 6.5mm FT CS for LM	49*	CAM	WBAT
3(L)	76	M	3	L	PM + LM	Enlocated	Closed	6.5mm FT CS for LM	49*	CAM	WBAT
4	89	F	1	R	MM + LM	Enlocated	Closed	6.5mm FT CS for LM; 6.5mm PT CS for MM	80	CAM	Touch WB
5(R)	92	F	5	R	Distal Tibia/fibula	N/A	Open	Tibial IMN; 6.5 FT CS for LM	145*	nil	WBAT
5(L)	92	F	5	L	Distal Tibia/fibula	N/A	Closed	Tibial IMN; 6.5 FT CS for LM	145*	nil	WBAT
6	71	F	3	R	MM+ LM	Enlocated	Closed	6.5mm FT CS for MM + LM	30	Cast	NWB
7	85	F	0	L	Trimalleolar	Dislocated	Closed	6.5mm FT CS for MM + LM; 4mm PT CS for PM	69	Cast	NWB
8	82	F	1	R	Trimalleolar	Subluxated	Closed	6.5mm FT CS for both MM + LM	48	CAM	WBAT
9	67	F	1	R	LM	Subluxated	Closed	6.5mm FT CS for LM + Suture Button for syndesmosis	39	Nil	WBAT
10	80	F	13	L	MM + LM	Subluxated	Closed	6.5mm FT CS for LM; 4mm PT CS for MM	75	Cast	NWB
11	89	F	1	R	Trimalleolar	Dislocated	Closed	6.5mm FT CS for MM + LM	50	Nil	WBAT

Case	Number of comorbidities	Pre op residential status	Pre op mobility	Latest mobility	Discharge destination	Clinical Frailty Score – On Admission	Clinical Frailty Score – On Discharge
1	2	Home	~~Nil aids~~Unaided	Unaided	Home	3	3
2	9	Home	Frame	Frame	Rehab	5	5
3(R)	10	Home	SPS	Steady Sara	Rehab	4	5
3(L)	10	Home	SPS	Steady Sara	Rehab	4	5
4	1	Home	~~Nil aids~~Unaided	Unaided	Rehab	3	4
5(R)	8	ACF	Frame	Steady Sara	ACF	6	7
5(L)	8	ACF	Frame	Steady Sara	ACF	6	7
6	11	Home	Frame	Steady Sara	Rehab	5	6
7	11	ACF	Frame	Frame	ACF	6	6
8	4	Home	~~Nil aids~~Unaided	Unaided	Rehab	3	3
9	8	Home	Frame	~~Unaided~~Frame	Rehab	4	4
10	4	Home	Frame	Frame	Home	4	4
11	5	ACF	Frame	Frame	ACF	5	5

Formatted Table

Case	Inpatient LOS (days)	Follow up duration (days)	Wound Complications	Non-wound complication
1	16	268	Small area slough at 6 weeks - managed with dressings only	Nil
2	3	3	Nil	Nil
3(R)	8	7	Nil	Nil
3(L)	8	7	Nil	Nil
4	5	87	Nil	Nil
5(R)	3	56	Nil	Nil
5(L)	3	56	Nil	Nil
6	0	8	Nil	Unrelated - death
7	20	114	nil	Nil
8	1	42	Nil	Nil
9	15	29	Abrasion from CAM boot - managed with dressings only	Deep infection of suture tape, re-operation for removal + debridement
10	7	38	Nil	Nil
11	3	42	Nil	Nil

Formatted Table
